# Dietary diversity and its association with changes in anthropometric indices of community-dwelling older adults in Tehran, Iran: a longitudinal study (2017–2021)

**DOI:** 10.1186/s12889-024-19635-y

**Published:** 2024-08-20

**Authors:** Mahshid Rezaei, Kimia Forouzan, Hassan Eini-Zinab, Nasrin Omidvar, Samaneh Jafaripour, Arezoo Rezazadeh

**Affiliations:** Department of Community Nutrition, Faculty of Nutrition Sciences and Food Technology, National Nutrition and Food Technology Research Institute, Shahid Beheshti University of Medical Sciences, Tehran, Iran

**Keywords:** Anthropometric indices, Diet, Dietary diversity score, Iran, Older adults

## Abstract

**Background:**

Dietary diversity refers to the consumption of a variety of foods or food groups over a given reference period, which is crucial for improving nutrition and overall health. This longitudinal study aimed to investigate the association between dietary diversity and anthropometric indices in community-dwelling older adults living in Tehran in 2017 and 2021.

**Methods:**

The current study was conducted on 368 older adults [204 (55.4%) women and 164 (44.6%) men] over 60 years of age living in Tehran, who were selected by a systematic cluster sampling method at two-time points, 2017 and 2021. Anthropometric measures (weight, height, hip circumference, and waist circumference) were assessed with standard methods. The participants’ dietary intake was assessed by completing two non-consecutive 24-hour recalls, and dietary diversity score (DDS) was calculated based on Kant’s method. Statistical analysis was performed using R software by the mixed effect model method.

**Results:**

The mean DDS of the participants in 2017 (5.07 ± 1.20) was higher than that in 2021 (4.94 ± 1.09) (*p* < 0.05). DDS and dairy diversity score decreased significantly over time. After adjusting for confounders, there was an inverse relationship between the DDS and Body Mass Index (BMI) (B = -0.22; ﻿SE = 0.09), but the interaction effect of year × DDS (B = 0.19; SE = 0.10﻿) was not significant (*p* = 0.06). However, there was a positive relationship between the DDS and A Body Shape Index (ABSI) (B = 0.00;﻿ p = 0.022), after adjusting for confounders, this relationship was no longer significant. Additionally, the interaction effect of year and DDS on the ABSI was not significant.

**Conclusion:**

The dietary intake and dietary diversity of older adult residents of Tehran declined dramatically with age, and a higher DDS was associated with improved anthropometric indices. DDS had an inverse relationship with general obesity in the studied participants, and the passage of time did not affect this relationship. The DDS can be used as a predictive index and is a powerful tool for investigating changes in nutritional status in longitudinal studies of old age. However, longer-duration studies are needed to obtain more conclusive results.

**Supplementary Information:**

The online version contains supplementary material available at 10.1186/s12889-024-19635-y.

## Background

In recent decades, factors such as socioeconomic developments, declining birth rates, increasing life expectancy, and access to healthcare services have led to a rise in the older adult population which is going to continue [1]. The World Health Organization (WHO) states that the older adult population will increase to 1.5 billion by 2050, and it is also predicted that their population will rise to 28% worldwide by the end of the 21st century [2, 3]. According to Iran’s national document of older adult individuals, the age of 60 is defined as the beginning of old age [1]. Iran has experienced one of the fastest rates of growth in the older adult population worldwide; the proportion of older adults in Iran increased from 4.2% in 1989 to 6.4% in 2019 [2]. In 2022, the proportion of older adults surpassed 10% of the total population, which is expected to reach one-third of Iran’s population in 2050 [4, 5]. Due to the growth of the old age population, paying attention to their health has become essential because they are more likely to suffer from different health issues and diseases, which can cause serious socioeconomic consequences if not taken into account [6]. One of the key factors affecting the well-being of older adult individuals is their diet and lifestyle [7]. With aging, due to physiological factors such as decreased appetite, loss of taste and smell, and a reduced ability to chew and swallow, older adults are at greater risk of malnutrition. As a result, this can influence their dietary intake, nutrient utilization efficiency, and nutritional status, leading to reduced dietary diversity [8–10].

Dietary intake involves consuming a diverse range of food items, each with intricate interactions between various nutrients. Analyzing single nutrients may potentially be confounded by the effect of overall diet. In this regard, investigation of dietary patterns shows a greater impact on health [11]. Dietary diversity is defined as consuming a different variety of food items or food groups over a given reference period [12]. Previous studies have shown inverse relationships between dietary diversity and obesity, blood pressure and cardiovascular diseases [13–15]. Dietary diversity score (DDS) is an appropriate and efficient tool for appraising dietary diversity in overall diet that is widely used across countries and all age groups [12, 16]. A higher DDS is associated with a better nutrient adequacy ratio and reflects diet quality, which will promote health. Increasing dietary diversity across and within food groups is recommended in most dietary guidelines [12]. Inadequate dietary diversity is a global problem. Consequently, assessing micronutrient adequacy and diet diversity in vulnerable populations is essential [17].

One important indicator of health in the older adult population is their anthropometric indices, which refer to the measurement of the body’s height, weight, and proportions of various body parts [18]. Anthropometry is a useful and easy-to-use tool that can provide valuable information about an individual’s health status, including functional status, nutrition and overall health [19].

Anthropometric indices could be influenced by individual’s nutritional status, and previous studies have shown conflicting results regarding the association between dietary diversity and changes in anthropometric measures [13, 20–24]. The results of previous cross-sectional studies could be inaccurate because variables that could change over time were excluded [25]. Moreover, the findings of studies that have presented models with time-independent variables have shown significantly different estimates and levels of significance compared to models that have presented time-dependent variables [26]. For greater accuracy in examining the relationship between exposure variables and outcomes, the linear mixed-effects model with a time variable can be used. A mixed effect model, by considering the impact of time and its confounding effect, could be used to predict the average change in the entire study sample in addition to within-individual changes [27]. This study, besides providing a nutritional database of older adults, can also be used for future comparison and determining the changes in the dietary intake of older adults over time, which can be used for identifying issues in nutritional planning. Thus, this longitudinal study aimed to investigate the association between dietary diversity and anthropometric indices in older adults living in Tehran in 2017 and 2021.

## Methods

### Study design and sampling

This longitudinal study is part of a broader study entitled “Situation Analysis of Free-living Elders’ Lifestyles (with an Emphasis on Nutrition)”. The present study was conducted on 368 older adults [204 (55.4%) women and 164 (44.6%) men] living in Tehran in two phases (2017 and 2021). According to the previous study, the sample size was calculated by Smee et al. [28] for community-dwelling older adults aged over 60 years. In the first phase of the study (2017), the research sample included 511 individuals chosen according to the inclusion criteria. The criteria included community-dwelling older adults over 60 years of age in Tehran, having Iranian citizenship, being willing to cooperate, having the ability to speak and communicate, lacking advanced diseases such as cancer and ESRD (End Stage Renal Disease), and lacking severe cognitive disorders such as Alzheimer’s disease and Parkinson’s disease. The exclusion criteria of this study included the older adult individuals who we didn’t have access to or who were not willing to complete and answer the 24-hour recall questionnaire, and the cases of over-reporting and under-reporting of energy below 500 and above 3500 kcal/day for women and below 800 and above 4000 kcal/day for men [29]. The population sampling method used was systematic cluster sampling, which considered the diverse socioeconomic statuses of residents of different geographical zones of Tehran, including the northern, southern, eastern, western, and central zones. Eleven municipal districts were selected across the zones, and based on population density, the number of older adults required for sampling was determined for each zone. From each municipal district, a health center [60% of the sample size], a nearby community center (*Saraye Mahalle*) [30% of the sample], and a mosque [10% of the sample] were randomly selected. All the individuals from the first phase were invited to participate in the second phase of the study. Considering the longitudinal design of the study and the expected dropouts, 375 individuals participated in 2021 with a 70% response rate and 368 individuals remained in the study after applying the exclusion criteria. To ensure the adequacy of the sample size of the study, a power analysis was calculated considering the result of a previous study on older adults by Karim Beigi et al. [30]. The power of the present study was calculated to be over 90%, which confirms the adequacy of the sample size (368 people) to obtain the expected results in this study. To ensure accurate and consistent data collection, a training session was held for nutritionists on how to use data collection techniques.

### Data measurement

In both phases of the study, the general information including demographic, socioeconomic, and lifestyle characteristics was collected through interviews with a valid questionnaire that had been used in previous studies [31, 32]. The questionnaire measured data based on sex, age group, marital status, ethnicity, educational level, family size, medications, supplements, income level, and the ratio of per capita food expenditure to per capita total cost.

### Assessment of dietary intakes

The participants’ dietary intake was assessed by completing two non-consecutive 24-hour recall questionnaires (a weekday and a weekend day), using the multiple-pass method [33], which has been applied in previous studies of Iranian older adults [34, 35]. This was completed through an in-person interview (on the first day) and a telephone interview (on the second day). In the dietary assessment interview, participants were prompted to recall their food and beverage intake for the previous day. During the interviews, participants were shown visual aids such as images of scales and measuring cups to help them remember the foods they had consumed accurately. Using Nutritionist IV software, the macronutrients and micronutrients of the food items were obtained. To calculate the energy and nutrient intake from other food items that were not available in the Nutritionist IV software, the food composition table (USDA, Release 11, 1994) was used and adapted for Iranian foods [36].

### Assessment of dietary diversity

The method described by Kant et al. [37] was utilized for determining dietary diversity. This method was based on 5 major groups comprising grains, vegetables, fruits, meats, and dairy products, and was based on the United States Department of Agriculture (USDA) food guide pyramid. The 5 main groups were also divided into 23 subgroups. The grain group was divided into 7 subgroups including refined bread, biscuits, macaroni, wholemeal bread, cornflakes, rice, and refined flour. The vegetable group was divided into 7 subgroups including green leafy vegetables, potato, tomato, other starchy vegetables, legumes, yellow vegetables, and other green vegetables. The fruit group was composed of 2 subgroups including fruit and fruit juice along with berries and citrus fruits. The meat group included 4 subgroups, such as red meat, poultry, fish, and eggs. Additionally, the dairy products were classified into 3 subgroups milk, yogurt (*Doogh and Kashk*), and cheese. If the participants ingested a minimum of half a unit from each subgroup within two days of recall, then they were allocated points for each group they consumed. The DDS for each main group was calculated by the division of an individual’s subgroup score by the total number of subgroups of that main group and multiplied by two. Eventually, the DDS was calculated from the sum of the diversity scores of all 5 major food groups, which ranged from a minimum of 0 to a maximum of 10 [30].

### Anthropometric measures

Anthropometric measures included weight (kg), height (cm), hip circumference (cm), and waist circumference (cm), which were measured using standard methods. The participant’s weight and waist circumference (distance around the smallest part of the waist, just above the umbilicus) were measured with accuracies of 100 g and 1 mm, respectively. Body mass index (BMI) was calculated based on weight in kilograms divided by the square of height in meters. Waist-to-hip ratio (WHR) and waist-to-height ratio (WHtR) were also calculated based on waist circumference divided by height. The calf muscle circumference (cm) (the thickest part of the calf without clothing) and the mid-upper arm circumference (distance between the acromion and the olecranon appendices) were measured using inflexible tape with an accuracy of 1 mm. As calf circumference and mid-upper arm circumference (cm) are strongly correlated with muscle mass, they have been proposed to be suitable indicators of muscle mass [38, 39]. Knee height (cm) was measured using a knee caliper with an accuracy of 5 mm. A body shape index (ABSI) was calculated as a complementary index to BMI to assess health risk based on waist circumference, height, and BMI using the following formula [40]. An ABSI greater than 0.083 was considered to indicate high ABSI and abdominal obesity [41].

### ABSI=$\:\frac{\text{W}\text{C}}{{\text{B}\text{M}\text{I}}^{2/3}\times\:{\text{H}\text{e}\text{i}\text{g}\text{h}\text{t}}^{1/2}}$

### Data analysis

After applying exclusion criteria, 368 older adult individuals over the age of 60 years remained for the final analysis. Eventually, the collected data were examined using IBM SPSS software (version 21.0) and R software (version 4.3.3). Once this process was completed, the Kolmogorov-Smirnov test assessed the distribution of quantitative data normality. To compare quantitative normal variables between two sex groups, the independent t-test was used, and the results were reported as the mean (standard deviation). For comparing non-normal quantitative variables, the median (interquartile range) was reported, and the Mann-Whitney test was used to compare determine the. When comparing non-normal quantitative variables, the Mann-Whitney test was used to determine the association between the variables and the reported median (interquartile range). The Wilcoxon test and paired t-test were used to compare variables between two time periods.

Using R software and the linear mixed effect model method, the relationship between anthropometric status and food diversity score was analyzed by including the effect of the year (time-variable) in the crude model (without adjusting the effect of confounders) and the adjusted model (adjusting the effect of all confounding variables entered in the model). For example, how the variables of the study are related can be explained by citing an example of the relationship between the DDS and BMI in two years:

BMI = β_0j_ + β_1_ year + β_2_ DDS + β_3_ year DDS + e_i_ β_0j_ = β_0_ + U_j_.

In this model, to check the effect of the DDS on BMI, a derivative must be taken:

$$\:\frac{{\Delta\:}\:\text{B}\text{M}\text{I}}{{\Delta\:}\:\text{D}\text{D}\text{S}}$$ = β_2_ + β_3_ year.

According to this equation, the effect of DDS on BMI is dependent on the study year, which means that DDS had an effect of β_2_ on BMI in 2017, but in 2021, this effect was β_2_ + β_3_. In the adjusted model, the method of calculation was the same, with the difference that the effect of confounding variables was also taken into account. With regard to this equation, the DDS effects rely on the year of the study. In other words, the DDS had an effect on β_2_ on BMI in the year 2017, while four years later, this effect was β_2_ + β_3_.

## Results

### Characteristics of the participants

The general characteristics of the participants are presented in Table 1. Of the 368 participants, 204 (55.4%) were women and 164 (44.6%) were men. The mean age was 67.06 ± 5.52 years and 70.89 ± 6.48 years in 2017 and 2021, respectively. Moreover, the percentage of individuals aged 60 to 64 years was 39.7% in 2017, the percentage of individuals aged 65 to 69 years was 37% in 2021, and the population of women under 70 years old was more than men in 2021. From 2017 to 2021, the proportion of older adult participants in the 60–64 years old age group decreased, and that of the older age groups increased. Considering the marital status in both study phases, the percentage of single, divorced, and widowed women was greater than men. In both phases, more participants lived as a couple, but the proportion of individuals living in a family of four or more decreased, and were moved to other categories of family size in 2021 compared to 2017. Women had lower incomes compared to men, and a greater percentage of older adults had average incomes in both years. The per capita total cost for men was higher than that of women in 2017. In 2021, there was a significant increase in the per capita total cost. Most of the participants did not have a university degree. The majority of the older adults were of Fars ethnicity (55.4%) and had less than a diploma education (62.8%). In both variables, the percentage of women was higher than men. Because the two variables of ethnicity and education level in 2021 were similar to those in 2017, these two variables have not been mentioned in Table 1. In 2017, women participants had more digestive problems than men did. Gastrointestinal disease incidence decreased in 2021 compared to 2017, and the use of blood pressure and lipid-lowering medication was also higher.

**Table 1:** General characteristics of the older adult population stratified by sex and comparisons between 2017 and 2021

Variable		2017 *n* (%)				2021 *n* (%)				*p*-value^d^
		Women	Men	Total	*p*-value^a^	Women	Men	Total	*p*-value^a^	
**Age (years)**	60–64	104 (51.0)	42 (25.6)	146 (39.7)	**< 0.01** ^***b***^	31 (15.2)	14 (8.5)	45 (12.2)	**< 0.01**	**< 0.01**
	65–69	62 (30.4)	47 (28.7)	109 (29.6)		92 (45.1)	44 (26.8)	136 (37.0)		
	70–74	28 (13.7)	41 (25.0)	69 (18.8)		51 (25.0)	38 (23.2)	89 (24.2)		
	75–79	8 (3.9)	27 (16.5)	35 (9.5)		20 (9.8)	43 (26.2)	63 (17.1)		
	≥ 80	2 (1.0)	7 (4.3)	9 (2.4)		10 (4.9)	25 (15.2)	35 (9.5)		
**Marital status**	Married	141 (69.1)	156 (95.1)	297 (80.7)	**< 0.01**	128 (62.7)	149 (90.9)	277 (75.3)	**< 0.01**	0.075
	Other	63 (30.9)	8 (4.9)	71 (19.3)		76 (37.3)	15 (9.1)	91 (24.7)		
**Family size**	Alone	24 (11.8)	7 (4.3)	31 (8.4)	**0.045**	33 (16.2)	7 (4.3)	40 (10.9)	**< 0.01**	**0.011**
	2	79 (38.7)	59 (36.2)	138 (37.6)		91 (44.6)	73 (44.5)	164 (44.6)		
	3	46 (22.5)	41 (25.2)	87 (23.7)		51 (25.0)	39 (23.8)	90 (24.5)		
	4	55 (27.0)	56 (34.4)	111 (30.2)		29 (14.2)	45 (27.4)	74 (20.1)		
**Taking medications**	Yes	183 (91.0)	134 (82.7)	317 (87.3)	**0.018**	185 (90.7)	135 (82.3)	320 (87.0)	**0.018**	0.913
	No	18 (9.0)	28 (17.3)	46 (12.7)		19 (9.3)	29 (17.7)	48 (13.0)		
**Taking Blood pressure medication**	Yes	86 (42.2)	69 (42.1)	155 (42.1)	0.987	113 (55.4)	78 (47.6)	191 (51.9)	0.135	**0.008**
	No	118 (57.8)	95 (57.9)	213 (57.9)		91 (44.6)	86 (52.4)	177 (48.1)		
**Taking Lipid-lowering medication**	Yes	97 (47.5)	51 (31.5)	148 (40.2)	**0.001**	119 (56.9)	60 (36.6)	176 (47.8)	**< 0.01**	**0.038**
	No	107 (52.2)	113 (68.9)	220 (59.8)		88 (43.1)	104 (63.4)	192 (52.2)		
**Taking diabetes medication**	Yes	54 (26.5)	37 (22.6)	91 (24.7)	0.388	56 (27.5)	47 (28.7)	103 (28.0)	0.798	0.315
	No	150 (73.5)	127 (77.4)	277 (75.3)		148 (72.5)	117 (71.3)	265 (72.0)		
**Taking Supplements**	Yes	156 (76.5)	62 (38.5)	218 (59.7)	**< 0.01**	165 (81.3)	76 (46.3)	241 (65.7)	**< 0.01**	0.091
	No	48 (23.5)	99 (61.5)	147 (40.3)		38 (18.7)	88 (53.7)	126 (34.3)		
**Gastrointestinal disease**	Yes	92 (45.1)	48 (29.3)	140 (38.0)	**0.002**	49 (24.0)	39 (23.8)	88 (23.9)	0.957	**< 0.01**
	No	112 (54.9)	116 (70.7)	228 (62.0)		155 (76.0)	125 (76.2)	280 (76.1)		
**Quantitative Variable**		**Women**	**Men**	**Total**		**Women**	**Men**	**Total**		**p-value** ^***d***^
		**Interquartile Range** **Middle**	**(IQR)** **Middle**	**(IQR)** **Middle**	**p-value** ^***c***^	**(IQR)** **Middle**	**(IQR)** **Middle**	**(IQR)** **Middle**	**p-value** ^***c***^	
**Per capita food cost/Per total cost (%)**		59.72 (47.66, 75)	66.66 (50, 79.16)	60 [50, 75]	**0.029**	66.66 [50, 80]	66.66 [50, 80]	66.66 [50, 80]	0.295	**0.002**

^*a*^ The *p*-value was obtained from the chi-square test for qualitative analysis

^***b***^ The Monte Carlo Exact test was used for this variable

^***c***^*P*-value for quantitative variables was performed based on the Mann-Whitney test and the results were reported as median (interquartile range (IQR)).

^*d*^ The *P*-value of comparing two years was reported based on the Wilcoxon test

### Anthropometric measures of the participants

The anthropometric measures and their classification of the participants in the two years of the study have been illustrated in Table 2. In both study phases, the average weight, height, WHR, and ABSI in men were higher than those in women. However, the average hip circumference, WHtR, mid-upper arm circumference, calf circumference, and BMI in women were higher than those in men. From 2017 to 2021, the average weight and calf circumference decreased, while the average WHtR increased. Based on a classification of anthropometric measures, no significant changes over time were observed. In both years, the proportion of individuals who were overweight or obese was higher, and there was a greater percentage of women in these groups than men. A larger ratio of women compared to men fell into the high-risk range for WHtR in both years. In 2017, a larger proportion of men participants compared to women fell into the high-risk range for WHR. Moreover, in both years, a higher proportion of men compared to women had a higher ABSI, placing them in the range of abdominal obesity.

**Table 2:** Anthropometric measures of the participants and their comparisons between 2017 and 2021

Variable		2017 *n* (%)				2021 *n* (%)					*p*-value^c^
		Women	Men	Total	*p*-value	Women	Men	Total	*p*-value		
**Height (cm)**		155.27 ± 6.23	167.49 ± 7.66	160.72 ± 9.19	**< 0.01** ^***a***^	154.69 ± 6.12	167.16 ± 7.08	160.25 ± 9.03	**< 0.01** ^***a***^		0.070
**Weight (kg)**		71.72 ± 11.51	74.52 ± 10.37	72.97 ± 11.09	**0.016** ^***a***^	70.88 ± 11.50	74.05 ± 10.32	72.29 ± 11.09	**0.006** ^***a***^		**0.001**
**Waist circumference (cm)**		96 (89.12, 105)	97 (90.12, 102.50)	97 (90, 104)	0.897^***b***^	98 (91, 105)	97.1 (91, 101.65)	97.35 (91, 103.85)		0.459^***b***^	0.508
**Hip circumference (cm)**		108.41 ± 11.63	100.38 ± 9.70	104.83 ± 11.51	**< 0.01** ^***a***^	107.98 ± 10.70	100.44 ± 9.31	104.62 ± 10.77	**< 0.01** ^***a***^		0.070
**Body mass index (kg/m**^**2**^)		29.69 (26.45, 32.74)	26.67 (23.84, 28.61)	27.76 (25.31, 30.77)	**< 0.01** ^***b***^	29.11 (24.08, 28.66)	26.60 (24.08, 28.66)	28.01 (25.18, 30.64)	**< 0.01** ^***b***^		0.656
**Classification of body mass index (BMI)**	Underweight	4 [2]	7 (4.3)	11 [3]	**< 0.01** ^***e***^	5 (2.5)	9 (5.5)	14 (3.8)	**< 0.01** ^***e***^		0.810
	Normal	55 [27]	84 (51.2)	139 (37.8)		50 (24.5)	85 (51.8)	135 (36.7)			
	Overweight and obese	145 [71]	73 (44.5)	218 (59.2)		149 [73]	70 (42.7)	219 (59.5)			
**Classification of waist circumference**	Optimal	88 (43.1)	59 [36]	147 (39.9)	0.163^***d***^	87 (42.6)	71 (43.3)	158 (42.9)	0.901^***d***^		0.410
	Risk	116 (56.9)	105 [64]	221 (60.1)		117 (57.4)	93 (56.7)	210 (57.1)			
**Classification of waist-to-height ratio (WHtR)**	Optimal	77 (37.7)	109 (66.5)	186 (50.5)	**< 0.01** ^***d***^	66 (32.4)	112 (68.3)	178 (48.4)	**< 0.01** ^***d***^		0.555
	Risk	127 (62.3)	55 (33.5)	182 (49.5)		138 (67.6)	52 (31.7)	190 (51.6)			
**Classification of Waist-to-hip ratio (WHR)**	Optimal	65 (31.9)	32 (19.5)	97 (26.4)	**0.008** ^***d***^	50 (24.5)	32 (19.5)	82 (22.3)	0.252^***d***^		0.197
	Risk	139 (68.1)	132 (80.5)	271 (73.6)		154 (75.5)	132 (80.5)	286 (77.7)			
**Classification of A Body Shape Index (ABSI)**	Optimal	133 (65.2)	68 (41.5)	201 (54.6)	**< 0.01** ^***d***^	121 (59.3)	60 (36.6)	181 (49.2)	**< 0.01** ^***d***^		0.140
	Abdominal obesity	71 (34.8)	96 (58.5)	167 (45.4)		83 (40.7)	104 (63.4)	181 (50.8)			

^***a***^ P-value was reported based on the independent sample T-test, and the results are reported as the mean ± SD.

^***b***^*P*-value was reported based on the Mann-Whitney test and the results were reported as median (interquartile range (IQR)).

^***c***^ The *P*-value comparing two years was reported based on the Wilcoxon test and paired sample T-test

^*d*^ The *p*-value was obtained from the chi-square test for qualitative analysis

^***e***^ The Monte Carlo Exact test was used for this variable

The normal BMI for older adults is 21–26.9 kg/m^2^. Values higher than this range were classified as overweight or obese, and values below this range were categorized as underweight

In men and women, a waist circumference less than 95 cm was considered the optimal limit

In men and women, a WHtR ≤ 0.6 was considered the optimal limit

In men, the WHR is less than 0.9, and in women, aWHR less than 0.85 was considered the optimal limit

An ABSI of more than 0.083 was considered to indicate a high-risk of abdominal obesity

### Energy intake status and DDS of participants

As shown in Table 3, during both phases of the study, the average energy intake of men was higher than women, and the average energy intake in 2017 was lower than that in 2021. The mean DDS was 5.07 ± 1.20 and 4.94 ± 1.09 in 2017 and 2021, respectively. The decrease in the DDS was considered almost significant (*p* = 0.054). In both phases of the study, men had a higher DDS than women, but this difference was more significant in 2021. In both phases, the meat diversity score of men was higher than that of women. In 2021, men also had a higher dairy diversity score compared to women. A comparison between the two years revealed that the dairy diversity score and the DDS decreased significantly.

**Table 3:** Energy intake and dietary diversity score (DDS) of participants and their comparison at two-time points

Variable	2017				2021				*p*-value^b^
	Women	Men	Total	*p*-value^a^	Women	Men	Total	*p*-value^a^	
**Energy (kcal)**	1348.90± 446.28	1673.26 ± 474.91	1493.45 ± 486.21	**< 0.01**	1215.59 ± 362.22	1466.24 ± 448.01	1327.29 ± 421.05	**< 0.01**	**< 0.01**
**Dietary Diversity Score (DDS)**	4.97 ± 1.26	5.19 ± 1.12	5.07 ± 1.20	0.080	4.79 ± 1.09	5.12 ± 1.06	4.94 ± 1.09	**0.004**	0.054
**Grains diversity score**	0.75 ± 0.22	0.77 ± 0.21	0.76 ± 0.21	0.456	0.80 ± 0.23	0.76 ± 0.20	0.78 ± 0.21	0.068	0.088
**Vegetables diversity score**	0.766 ± 0.327	0.815 ± 0.347	0.788 ± 0.336	0.164	0.798 ± 0.327	0.799 ± 0.327	0.798 ± 0.327	0.977	0.622
**Fruits diversity score**	1.55 ± 0.62	1.56 ± 0.61	1.55 ± 0.61	0.914	1.50 ± 0.60	1.57 ± 0.57	1.53 ± 0.59	0.251	0.615
**Meat diversity score**	0.76 ± 0.38	0.85 ± 0.43	0.80 ± 0.41	**0.032**	0.74 ± 0.41	0.86 ± 0.42	0.79 ± 0.42	**0.005**	0.730
**Dairy diversity score**	1.13 ± 0.59	1.18 ± 0.50	1.15 ± 0.55	0.328	0.94 ± 0.52	1.11 ± 0.47	1.01 ± 0.51	**0.001**	**< 0.01**

^*a*^ It was reported﻿ based on an independent sample T-test, and the results are reported as the mean ± SD.

^*b*^ It was reported based on the paired sample T-test

### Associations between DDS and anthropometric measures

The association between DDS and anthropometric measures of the participants is presented in Tables 4 and 5. According to both crude and adjusted models, there was an inverse relationship between the DDS and BMI. According to the crude model, for every unit increase in the DDS, BMI decreased by 0.27 units (*p* = 0.001). Considering the longitudinal nature of the study, the interaction effect of DDS on BMI (year × DDS) was 0.188 between 2017 and 2021. Due to the decrease of this negative relationship by 0.188, the interaction effect was not significant (*p* = 0.06). In the adjusted model, there was an inverse relationship between DDS and BMI. Additionally, the discrepancy in the effect of DDS on BMI between the two years was not as significant as in the crude model (Table 4).

**Table 4:** Association of dietary diversity score with BMI, and waist circumference of participants (considering the effect of the year): mixed-effects model

			BMI						Waist circumference					
			Crude Model			Adjusted model			Crude Model			Adjusted model		
			Coefficients	Standard error	*p*-value	Coefficients	Standard error	*p*-value	Coefficients	Standard error	*p*-value	Coefficients	Standard error	*p*-value
Grains diversity score	**Fixed effect**	Intercept elevation	28.329	0.406	< 0.01	26.421	2.854	< 0.01	95.912	1.031	< 0.01	97.428	7.427	< 0.01
		Year	-0.764	0.459	0.097	-0.585	0.488	0.231	-0.621	1.181	0.598	-1.463	1.260	0.246
		Grains	0.045	0.438	0.917	0.213	0.447	0.633	0.680	1.127	0.546	0.512	1.156	0.657
		Year × Grains	0.769	0.577	0.183	0.582	0.581	0.317	1.235	1.485	0.406	1.737	1.501	0.248
	Random effect (standard deviation)	Intercept elevation	4.220			3.893			10.338			10.199		
		Residual	1.323			1.319			3.408			3.401		
**Vegetables diversity score**	**Fixed effect**	Intercept elevation	28.511	0.324	< 0.01	26.554	2.863	< 0.01	96.173	0.814	< 0.01	97.896	7.444	< 0.01
		Year	-0.198	0.314	0.528	-0.133	0.348	0.702	-0.058	0.810	0.942	-0.693	0.901	0.441
		Vegetables	-0.186	0.288	0.518	-0.084	0.295	0.775	0.329	0.741	0.657	0.202	0.762	0.791
		Year × Vegetables	0.056	0.377	0.880	0.059	0.382	0.877	0.533	0.970	0.582	0.837	0.987	0.397
	Random effect (standard deviation)	Intercept elevation	4.227			3.897			10.332			10.186		
		Residual	1.326			1.323			3.417			3.415		
**Fruits diversity score**	**Fixed effect**	Intercept elevation	28.791	0.339	< 0.01	26.730	2.855	< 0.01	96.508	0.855	< 0.01	97.598	7.443	< 0.01
		Year	-0.329	0.322	0.307	-0.246	0.347	0.479	0.715	0.832	0.390	0.256	0.901	0.776
		Fruits	-0.274	0.159	0.086	-0.241	0.161	0.134	-0.048	0.411	0.906	-0.054	0.417	0.895
		Year × Fruits	0.109	0.198	0.581	0.103	0.201	0.606	-0.224	0.512	0.661	-0.167	0.522	0.748
	Random effect (standard deviation)	Intercept elevation	4.217			3.890			10.324			10.190		
		Residual	1.324			1.321			3.424			3.420		
**Meat diversity score**	**Fixed effect**	Intercept elevation	28.757	0.298	< 0.01	26.733	2.865	< 0.01	96.335	0.749	< 0.01	97.240	7.463	< 0.01
		Year	-0.659	0.258	0.011	-0.579	0.299	0.053	-0.286	0.665	0.667	-0.834	0.775	0.282
		Meats	-0.486	0.235	0.039	-0.317	0.241	0.189	0.120	0.605	0.842	-0.042	0.624	0.945
		Year × Meats	0.625	0.297	0.036	0.596	0.299	0.056	0.824	0.766	0.283	0.997	0.774	0.198
	Random effect (standard deviation)	Intercept elevation	4.221			3.895			10.339			10.196		
		Residual	1.319			1.317			3.410			3.408		
**Dairy diversity score**	**Fixed effect**	Intercept elevation	29.056	0.308	< 0.01	27.025	2.851	< 0.01	96.530	0.779	< 0.01	97.736	7.452	< 0.01
		Year	-0.588	0.257	0.022	-0.488	0.287	0.089	0.433	0.671	0.518	0.003	0.749	0.996
		Dairy	-0.598	0.177	< 0.01	-0.518	0.184	0.005	-0.084	0.462	0.855	-0.225	0.481	0.639
		Year × Dairy	0.343	0.220	0.120	0.354	0.221	0.111	-0.073	0.575	0.898	-0.017	0.579	0.975
	Random effect (standard deviation)	Intercept elevation	4.213			3.893			10.324			10.189		
		Residual	1.311			1.311			3.425			3.420		
**Dietary Diversity Score (DDS)**	**Fixed effect**	Intercept elevation	29.750	0.480	< 0.01	27.513	2.869	< 0.01	96.014	1.232	< 0.01	97.722	7.497	< 0.01
		Year	-1.120	0.515	0.030	-1.035	0.534	0.053	-0.241	1.336	0.856	-1.010	1.389	0.467
		DDS	-0.273	0.083	0.001	-0.226	0.090	0.012	0.082	0.215	0.702	0.030	0.234	0.895
		Year × DDS	0.188	0.101	0.066	0.193	0.102	0.060	0.126	0.262	0.631	0.202	0.265	0.445
	Random effect (standard deviation)	Intercept elevation	4.205			3.886			10.346			10.201		
		Residual	1.314			1.315			3.415			3.414		

BMI (body mass index) and waist circumference were considered dependent variables, and dietary diversity score was consider an independent variable

The year indicated in the table refers to 2021

In the adjusted model, the effects of variables such as age, sex, energy, education level, family size, medication consumption (blood pressure, lipid-lowering, diabetes), gastrointestinal disease, supplements, and the ratio of per capita food expenditure to per capita total cost were adjusted

**Table 5:** Association of dietary diversity score with WHR and ABSI of participants (considering the effect of the year): mixed-effects model

			WHR						ABSI					
			Crude Model			Adjusted model			Crude Model			Adjusted model		
			Coefficients	Standard error	*p*-value	Coefficients	Standard error	*p*-value	Coefficients	Standard error	*p*-value	Coefficients	Standard error	*p*-value
**Grains diversity score**	**Fixed effect**	Intercept elevation	0.931	0.013	< 0.01	1.066	0.076	< 0.01	0.081	0.000	< 0.01	0.078	0.004	< 0.01
		Year	-0.000	0.017	0.964	-0.010	0.018	0.570	0.001	0.000	0.108	0.000	0.001	0.889
		Grains	-0.008	0.016	0.587	-0.015	0.016	0.361	0.000	0.000	0.566	0.000	0.000	0.876
		Year × Grains	0.006	0.021	0.766	0.017	0.021	0.423	-0.001	0.001	0.391	0.000	0.001	0.876
	Random effect (standard deviation)	Intercept elevation	0.090			0.082			0.005			0.005		
		Residual	0.051			0.051			0.002			0.002		
**Vegetables diversity score**	**Fixed effect**	Intercept elevation	0.917	0.010	< 0.01	1.056	0.076	< 0.01	0.081	0.000	< 0.01	0.078	0.004	< 0.01
		Year	0.009	0.011	0.404	0.004	0.012	0.702	0.001	0.000	0.133	0.000	0.000	0.972
		Vegetables	0.009	0.010	0.359	0.007	0.010	0.485	0.000	0.000	0.162	0.000	0.000	0.242
		Year × Vegetables	-0.007	0.014	0.602	-0.002	0.014	0.854	0.000	0.000	0.682	0.000	0.000	0.977
	Random effect (standard deviation)	Intercept elevation	0.090			0.828			0.005			0.005		
		Residual	0.051			0.051			0.002			0.002		
**Fruits diversity score**	**Fixed effect**	Intercept elevation	0.934	0.010	< 0.01	1.066	0.076	< 0.01	0.082	0.000	< 0.01	0.078	0.004	< 0.01
		Year	0.000	0.012	0.994	-0.001	0.012	0.893	0.001	0.000	0.099	0.000	0.000	0.614
		Fruits	-0.006	0.005	0.293	-0.006	0.005	0.287	0.000	0.000	0.843	0.000	0.000	0.885
		Year × Fruits	0.002	0.007	0.736	0.003	0.007	0.678	0.000	0.000	0.555	0.000	0.000	0.571
	Random effect (standard deviation)	Intercept elevation	0.090			0.082			0.005			0.005		
		Residual	0.051			0.051			0.002			0.002		
**Meat diversity score**	**Fixed effect**	Intercept elevation	0.915	0.008	< 0.01	1.055	0.076	< 0.01	0.081	0.000	< 0.01	0.077	0.004	< 0.01
		Year	0.020	0.009	0.035	0.019	0.010	0.073	0.001	0.000	0.045	0.000	0.000	0.812
		Meats	0.011	0.008	0.174	0.007	0.008	0.382	0.000	0.000	0.077	0.000	0.000	0.280
		Year × Meats	-0.020	0.011	0.068	-0.019	0.011	0.081	0.000	0.000	0.497	0.000	0.000	0.750
	Random effect (standard deviation)	Intercept elevation	0.090			0.083			0.005			0.005		
		Residual	0.051			0.051			0.002			0.002		
**Dairy diversity score**	**Fixed effect**	Intercept elevation	0.911	0.009	< 0.01	1.049	0.076	< 0.01	0.081	0.000	< 0.01	0.077	0.004	< 0.01
		Year	0.023	0.009	0.016	0.023	0.010	0.028	0.001	0.000	0.001	0.001	0.000	0.088
		Dairy	0.012	0.006	0.068	0.011	0.006	0.102	0.000	0.000	0.027	0.000	0.000	0.099
		Year × Dairy	-0.017	0.008	0.077	-0.018	0.008	0.088	0.000	0.000	0.054	0.000	0.000	0.048
	Random effect (standard deviation)	Intercept elevation	0.090			0.082			0.005			0.005		
		Residual	0.051			0.051			0.002			0.002		
**Dietary Diversity Score (DDS)**	**Fixed effect**	Intercept elevation	0.912	0.016	< 0.01	1.052	0.077	< 0.01	0.080	0.000	< 0.01	0.077	0.004	< 0.01
		Year	0.033	0.019	0.090	0.029	0.020	0.149	0.002	0.001	0.025	0.001	0.001	0.220
		DDS	0.002	0.003	0.406	0.001	0.003	0.694	0.000	0.000	0.022	0.000	0.000	0.093
		Year × DDS	-0.005	0.003	0.130	-0.005	0.003	0.181	0.000	0.000	0.120	0.000	0.000	0.198
	Random effect (standard deviation)	Intercept elevation	0.090			0.083			0.005			0.005		
		Residual	0.051			0.051			0.002			0.002		

In this regard, WHR (waist-hip ratio) and ABSI (a body shape index) were considered dependent variables, and dietary diversity was an independent variable

The year indicated in this table refers to 2021

In the adjusted model, the effects of variables such as age, sex, energy, education level, family size, medication consumption (blood pressure, lipid-lowering, diabetes), gastrointestinal disease, supplements, and the ratio of per capita food expenditure to per capita total cost were adjusted

An inverse relationship between dairy diversity score and BMI existed in both models. According to the Crude model, for every 1 unit increase in dairy diversity score, BMI decreased by 0.059 units (*p* < 0.01). However, the effect of the dairy diversity score on BMI did not differ significantly between the two years (*p* = 0.120). According to the adjusted model, there was an inverse relationship between the dairy diversity score and BMI (*p* = 0.005), but the difference in the effect between the two years remained non-significant (*p* = 0.111) (Table 4). There was no significant relationship between the DDS and its components with waist circumference, WHR, and WHtR. There was a positive relationship between DDS and ABSI (*p* = 0.022). However, after adjusting for confounders, this relationship was no longer significant. In both models, the interaction effect of year and DDS on ABSI was not significant (Table 5).

## Discussion

To the best of our knowledge, this is the first longitudinal study that investigated the association between dietary diversity and anthropometric indices among Iranian older adults. This findings revealed that most of the participants had moderate dietary diversity in both 2017 and 2021, and DDS decreased over time. After adjusting for possible confounders, DDS and BMI had an inverse relationship. However, this association did not significantly change over time. The participants’ anthropometric indices, including BMI, WHtR, and WHR, were above the normal range in both 2017 and 2021 based on the cutoff values for the older adult population. In addition, while the average weight, mid-upper arm circumference, and calf circumference decreased, the average WHtR increased over time.

According to the findings, there was moderate dietary diversity in the older adult population in both years [42]. No longitudinal study has investigated the changes in DDS in Iranian older adults, however, according to the results of a cross-sectional study on the older adult women in Tehran with a mean age of 67.1 ± 4.8, the mean DDS (4.22 ± 1.28) is lower than the DDS reported in the present study [43]. This discrepancy may be due to the smaller sample size (*n* = 300) which included only women participants and a different method for calculating DDS was used. Likewise, in a study on Taiwanese older adults, the mean of the DDS (4.74 ± 0.97) was lower than the score reported in the current study, and participants with a lower DDS had consumed less dairy, vegetables and fruits [44]. One of the reasons behind the findings of this study with the present study can be the use of a food frequency questionnaire (FFQ) in their study. Similar to the present study, a longitudinal study with 12 years follow-up on Japanese older adult individuals (NILS-LSA) found that the fixed effects of interaction between age and time on the change of DDS were significant and DDS decreased in participants aged 63 to 79 years [45]. Previous studies have shown that higher dietary diversity is associated with better nutritional status in older adults [46]. Consequently, a reduction in food intake (energy and macronutrients) may be one of the potential causes of the decrease in the current study’s DDS. With aging, food intake decreases especially for fresh fruit and vegetables which is due to physiological changes such as lower chewing ability because of the lack of teeth or use of artificial teeth, some problems in swallowing and indigestion, and chronic diseases [47]. Sociodemographic factors such as educational level and marital status also can affect dietary diversity [48–50]. In this regard, a study on Thai older adults showed that a higher dietary diversity was associated with a higher educational level [48]. In addition, the ability of older people to perform daily life activities declines with aging [51]. In this regard, the possibility of daily activities such as shopping or preparing food is probably more difficult for this age group and can lead to a decline in dietary diversity.

One of the major components of the DDS, the dairy diversity score, experienced a significant decline over the study period. This decrease in dairy consumption was also observed in previous studies of Iranian households [52–54]. Dairy products are essential for preventing bone loss and reducing fracture risk in older adults, as they include energy, carbohydrates, cholesterol, vitamins, and riboflavin and are rich in protein and calcium [55, 56]. Nevertheless, the decline in participants’ dairy diversity was mainly because of rising inflation in Iran resulting from international sanctions, which effectively reduced the number of goods that could be purchased with a given amount of money [55]. Additionally, the lower consumption of dairy products in older adults may be attributed to digestive side effects of the lactose in dairy products, such as bloating, abdominal pain, flatulence, and diarrhea [56].

In this study, there was an inverse relationship between the DDS and BMI, and over time, the attenuating effect of this relationship was not considered to be statistically significant. Findings from other cross-sectional studies on adults and older adults have sometimes been contradictory [13, 23] or similar [22, 24] to the current study. In addition, several studies have found no significant relationship between DDS and BMI [21, 57–59]. A reason for differences in the results of these studies could be because of using different methods for the calculation of DDS. In the present study, the method for calculating DDS was based on the approach by Kant, which includes the five main food groups from the food pyramid (grains, fruits, vegetables, meat, and dairy products) along with 23 sub-groupings and did not include sweetened beverages, sweets, nuts, and fats [37]. Some studies reporting a positive relationship between dietary diversity and BMI have demonstrated that a higher level of dietary diversity might result in higher food consumption and additional energy intake, especially among middle-aged people, which subsequently can cause weight gain [60, 61]. Furthermore, studies conducted on different age profiles, like children and adolescents, have illustrated that a lower level of dietary diversity is linked to changes in weight, anthropometric measures, and body composition over time [20]. The variability in study outcomes could be due to differences in the calculation of the DDS.

Although an inverse relationship was observed between the dairy diversity score and BMI that began to decrease with time during the study, the effect of this relationship was not statistically significant. Various cross-sectional and cohort studies in adult and older adult populations have confirmed a negative relationship between dairy consumption and obesity [62–65]. Different mechanisms have been suggested to elaborate on how dairy may affect body composition. Dairy products are important sources of calcium, vitamin D, and protein, which can reduce the obesity rate [66, 67]. It has also been proposed that milk is rich in bioactive peptides that may play a significant role in regulating body fat accumulation [68]. In addition, milk contains various hormones and growth factors derived from the bovine animal which are similar to those found in humans [69]. Although many hormones are metabolized or broken down during digestion, intact hormones that are absorbed may have potential effects on metabolism [69]. Although we adjusted possible confounders, especially energy and age variables in the adjusted model in the present study, we did not consider the fat content in dairy products, and we didn’t separate dairy products into high-fat and low-fat groups that can be a reason for differences in results. Some studies have found that the fat content of dairy products can be a potential factor in explaining the relationship between dairy diversity score and BMI [62, 70]. This distinction may explain differences between our results and those of other studies.

After adjustment for all potential confounders, the findings of this study suggested that there was no significant association between DDS and ABSI. Additionally, the interaction effect of year and DDS on the ABSI was not significant. This result could be due to participants having an overall adequate intake of nutrients, regardless of dietary diversity, which could mask the potential effects of DDS changes on ABSI. In addition, in the older adult population due to the increase in sarcopenic obesity related to aging, the ABSI, along with other anthropometric measures, can provide desirable results [71]. Since our study was not adjusted for sarcopenic obesity, this factor may have contributed to the non-significant association observed. The majority of previous studies have concentrated on the relationship between ABSI and diseases and mortality, instead of its association with dietary diversity [72–75]. Only one longitudinal study, conducted in Indonesia from 2007 to 2014, examined the relationship between dietary security and ABSI among middle-aged adults and found an inverse relationship between dietary security and ABSI [76]. However, a direct comparison between these two studies may not be feasible due to differences in the exposure variables. One of the advantages of the ABSI is that it combines information on waist circumference in addition to height and weight [77]. A high ABSI indicates a proportionally higher than expected waist circumference for a given height and weight, which be related to greater central fat accumulation. The ABSI independently predicts mortality, independent of BMI [78]. In the DECODE study, a positive linear correlation was observed between CVD-related mortality and ABSI, whereas BMI, WC, and WHR displayed J-shaped relationships [79].

The study findings revealed that the older adult participants were in the overweight or obese range based on their BMI in both years of the study. Additionally, their waist circumference and WHR indicated an increased risk for health problems. Although there was a decrease in the average weight and the mid-upper arm circumference and calves in 2021 compared to those in 2017, the average WHtR increased. Conversely, in the only longitudinal study conducted on the Iranian older adult population in Babol [80], after 5 years (2011–2016), a significant increase in BMI was observed. However, height, waist circumference, hip circumference, WHR, and WHtR significantly decreased. The decrease in weight was not statistically significant. The larger sample size (*n* = 897) in the Babol study or the use of different measurement tools caused the difference in the measured variables in this study compared to the current study. However, other longitudinal studies conducted in different countries have reported results similar to our findings [81, 82]. For instance, in a study in Sweden [81], the calf and mid-upper arm circumferences decreased significantly after 15 years. The significant decrease in calf and mid-upper arm circumference, coupled with the increase in WHtR, suggests a decrease in muscle mass and an increase in body fat, especially in the central areas of older adult individuals. Several studies agree on the aforementioned issue [83–86]. This can be attributed to several factors, including increased age, and physiological changes such as sarcopenic obesity, decreased physical activity due to limitations such as air pollution, the industrialized nature of life, and the presence of diseases like COVID-19 in recent years [87, 88]. Another factor that can influence this issue is nutritional factors. In the current study, there was a decrease in the average energy and protein intake of the older adult participants over time, which is consistent with previous findings that an inadequate intake of energy and protein can result in a reduction in muscle mass and the progression of sarcopenia [89–91].

According to the assessment of participants’ dietary intake, their average daily energy intake was below the recommended dietary allowance (RDA) for both men and women in both years of the study [92]. Some studies conducted on older adults in Iran and other countries have also reported low energy intake in older adults [93–95]. According to a study conducted on Sabzevar’s institutionalized seniors, the average energy intake of participants was also lower than RDA values (1659.68 ± 497.94) [93]. Similarly, a study conducted on individuals aged over 60 years in Babol revealed that daily energy intake based on data obtained from two questionnaires (the FFQ (1535.4 kcal/day) and 24-hour recall intake questionnaire (1470.2 kcal/day)) was below the RDA values [94]. The energy intake of participants declined due to decreased food consumption such as fiber-rich vegetables, whole grains, and nuts in Babol’s study. This state was related to their dental problems, chewing difficulties, medication side effects, or declining mental and physical health. Another study on 217 older adult women aged 70–80 years in Australia also showed lower energy intake than RDA values (1450.1 kcal/day) [95]. One of the reasons for this decreased energy intake may be explained by physiological changes with aging such as decreased appetite, loss of taste and smell, oral and dental problems, delayed gastric emptying, altered hormonal responses, and reduction of basal metabolic rate [96]. Furthermore, under-reporting of energy intake and socioeconomic problems can be other reasons for the decreased energy intake in this study. In this regard, a study in the United Kingdom showed that older adults who lived alone felt that eating alone was less enjoyable than eating with others, and they often bought less food [97].

The main strength of this study was that it focused on the community-dwelling older adult population instead of on institutionalized seniors. Another advantage of this study was the generalizability of the selected older adults to the entire older adult population of Tehran due to sampling from all zones of Tehran and various settings including health centers, mosques, and *Saraye Mahalleh*. The longitudinal nature of the study design provided the possibility of examining the changes in the examined variables over time. The linear mixed effect model provides a more valid and accurate estimate for examining the relationship between the exposure variables and the outcome. Another positive feature of this approach is that it takes residuals and time effects into account.

However, the current study has not been without limitations. One constraint of the study was the smaller sample size in 2021 compared to 2017. The number of participants in the second phase of the study was lower due to concerns about the spread of COVID-19. Additionally, there were difficulties in reconnecting with participants who had changed their contact information. However, the study had high statistical power despite sample attrition. Furthermore, when assessing the dietary intake of older adult individuals through 24-hour recall, sources of bias were identified. For example, some older adult individuals, especially men, experienced difficulty recalling their previous day’s dietary intake, and some older people had trouble remembering the ingredients of mixed dishes. To minimize recall bias, the participants’ dietary intake information was validated by their spouse or a family member who had frequent contact with them.

## Conclusion

In conclusion, the findings suggested that dietary intake and dietary diversity in older adult residents of Tehran declined dramatically with age, and a higher DDS was associated with improved anthropometric indices. DDS had an inverse relationship with general obesity in the community living older adults of Tehran. Furthermore, the passage of time did not affect this relationship. Therefore, DDS can be used as a predictive index and is a powerful tool for investigating changes in nutritional status in longitudinal studies for older adults. However, to obtain more conclusive results, longer-duration studies are recommended.

### Electronic supplementary material

Below is the link to the electronic supplementary material.

Supplementary Material 1

## Data Availability

The datasets obtained and/or analyzed during the current study are not publicly available as the datasets are highly detailed and we are planning to publish more papers using the same dataset but are available from the corresponding author upon request.

## Abbreviations

ABSI: A Body Shape Index
BMI: Body Mass Index
CVD: Cardiovascular diseases
DDS: Dietary Diversity Score
DECODE: Diabetes Epidemiology Collaborative Analysis of Diagnostic Criteria in Europe
ESRD: End Stage Renal Disease
FFQ: Food Frequency Questionnaire
RDA: Recommended Dietary Allowance
SD: Standard Deviation
USDA: United States Department of Agriculture
WC: Waist Circumference
WHO: World Health Organization
WHR: Waist-to-Hip Ratio
WHtR: Waist-to-Height Ratio
