# Supplementary material for: Dietary diversity and its association with changes in anthropometric indices of community-dwelling older adults in Tehran, Iran: a longitudinal study (2017–2021)
Source: BMC Public Health. 2024 Aug 20;24:2253. doi: 10.1186/s12889-024-19635-y (PMC11334311; doi:10.1186/s12889-024-19635-y)
Supplement: Supplementary file 1 — Supplementary Material 1 [file 12889_2024_19635_MOESM1_ESM.pdf]

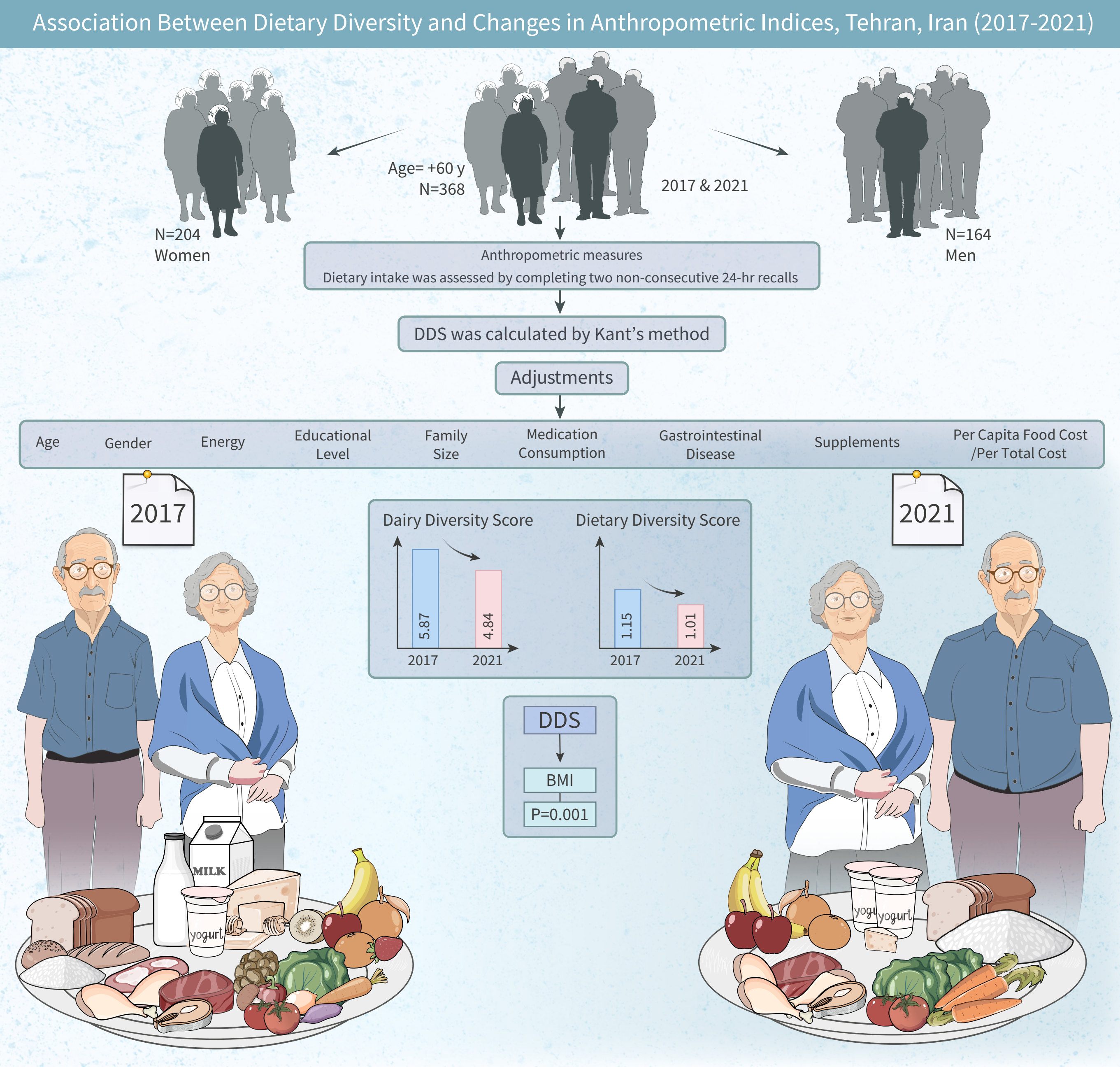
Association Between Dietary Diversity and Changes in Anthropometric Indices, Tehran, Iran (2017-2021)
Age= +60 y
N=368
2017 & 2021
N=204
Women
N=164
Men
Anthropometric measures
Dietary intake was assessed by completing two non-consecutive 24-hr recalls
DDS was calculated by Kant's method
Adjustments
Age
Gender
Energy
Educational Level
Family Size
Medication Consumption
Gastrointestinal Disease
Supplements
Per Capita Food Cost /Per Total Cost
2017
2021
Dairy Diversity Score
5.87
4.84
2017
2021
Dietary Diversity Score
1.15
1.01
2017
2021
DDS
BMI
P=0.001
MILK
yogurt
yogurt
yogurt
